# Crosstalk between Statins and Cancer Prevention and Therapy: An Update

**DOI:** 10.3390/ph14121220

**Published:** 2021-11-25

**Authors:** Beniamin Oskar Grabarek, Dariusz Boroń, Emilia Morawiec, Piotr Michalski, Veronica Palazzo-Michalska, Łukasz Pach, Barbara Dziuk, Magdalena Świder, Nikola Zmarzły

**Affiliations:** 1 Department of Histology, Cytophysiology and Embryology, Faculty of Medicine, University of Technology in Katowice, 41-800 Zabrze, Poland; dariusz@boron.pl (D.B.); emilia.wojdas@gmail.com (E.M.); barbaradziuk@poczta.onet.pl (B.D.); malena72@gmail.com (M.Ś.); nikola.zmarzly@gmail.com (N.Z.); 2 Department of Gynecology and Obstetrics, Faculty of Medicine, University of Technology in Katowice, 41-800 Zabrze, Poland; 3 Department of Gynecology and Obstetrics with Gynecologic Oncology, Ludwik Rydygier Memorial Specialized Hospital, 31-826 Kraków, Poland; 4 Departament of Gynecology and Obstetrics, TOMMED Specjalisci od Zdrowia, 40-662 Katowice, Poland; 5 Department of Microbiology, Faculty of Medicine, University of Technology in Katowice, 41-800 Zabrze, Poland; 6 Gyncentrum, Laboratory of Molecular Biology and Virology, 40-851 Katowice, Poland; 7 Department of Pathophysiology, Faculty of Medicine, University of Technology in Katowice, 41-800 Zabrze, Poland; piotrm703@gmail.com (P.M.); ver.palazzo@gmail.com (V.P.-M.); 8 Voivodeship Emergency in Katowice, 40-055 Katowice, Poland; lukpach@wp.pl

**Keywords:** statins, cancer, cancer prevention, anticancer therapy

## Abstract

The importance of statins in cancer has been discussed in many studies. They are known for their anticancer properties against solid tumors of the liver or lung, as well as diffuse cancers, such as multiple myeloma or leukemia. Currently, the most commonly used statins are simvastatin, rosuvastatin and atorvastatin. The anti-tumor activity of statins is largely related to their ability to induce apoptosis by targeting cancer cells with high selectivity. Statins are also involved in the regulation of the histone acetylation level, the disturbance of which can lead to abnormal activity of genes involved in the regulation of proliferation, differentiation and apoptosis. As a result, tumor growth and its invasion may be promoted, which is associated with a poor prognosis. High levels of histone deacetylases are observed in many cancers; therefore, one of the therapeutic strategies is to use their inhibitors. Combining statins with histone deacetylase inhibitors can induce a synergistic anticancer effect.

## 1. Introduction

3-Hydroxy-3-methylglutaryl coenzyme A (HMG-CoA) reductase inhibitors, known as statins, are a commonly used and well-tolerated class of drugs used in lipid disorders, especially in cases of hypercholesterolemia. Their effectiveness in preventing the development of cardiovascular diseases makes statins one of the most widely used drugs [1]. While there are clear benefits of statins in reducing mortality in high-risk patients, it is still uncertain whether statins may increase or decrease the risk of cancer [2]. The effect of long-term statin use is quite complex, as they have multiple properties that go beyond lipid lowering. There is evidence that statins exacerbate endothelial dysfunction and decrease levels of inflammatory markers [3,4]; however, it is still unclear whether they influence cancer risk. The studies conducted so far have shown, on the one hand, the carcinogenic effect of statins [5], and, on the other hand, no effect on carcinogenesis [6] or even their protective effect [7,8].

There are currently many observational studies available that aim to assess the risk of individual cancers in long-term statin users. However, only some studies have found that statin use has lasted longer than 5 years [9]. None of these findings are statistically significant, except in one study showing a reduced risk of prostate cancer in 42 statin users [10]. In addition, it has been shown that over five years of statin use in 10 common cancers, including prostate, breast, colorectal, lung, bladder, pancreatic, renal cell, melanoma, endometrial cancer and non-Hodgkin lymphoma, significantly reduced the risk of hematological cancers and increased endometrial cancer risk associated with more than 5 years of use of statins [11].

The aim of this paper is to present the current state of knowledge on the potential mechanisms of statin action in reducing the risk of cancer and its therapy.

## 2. Cholesterol Biosynthesis, Statin Structure and Their Applications

Cholesterol is one of the basic building elements of cell membranes in the human body, as well as the substrate for steroid hormone production. Its synthesis under physiological conditions is regulated at many levels; however, the key point is the enzymatic conversion of HMG-CoA to mevalonate, catalyzed by HMG-CoA reductase. Physiologically, this reaction can be limited by mevalonate derivatives and sterol regulatory element-binding proteins (SREBPs) [12]. Overexpression of the cholesterol biosynthesis pathway genes in cancer cells leads to a more severe course of neoplastic disease and is associated with resistance to treatment. Thus, the cholesterol biosynthetic pathway may be a potential target in anti-cancer therapy. However, it seems that a high dose of statin is needed, e.g., at least 60 mg/day in the case of simvastatin [13,14].

Statins are one of the key drugs used in the course of hypercholesterolemia, the first of which is lovastatin, which was introduced to the market in 1987 [15]. Nowadays, mainly simvastatin, rosuvastatin and atorvastatin are used. The oldest and best-studied of them is simvastatin, but rosuvastatin is characterized by the greatest potency in the percentage reduction in low density lipoprotein (LDL), so it should be selected as the lipid-lowering drug of first choice in patients with cardiovascular diseases [16]. On the other hand, atorvastatin should be used in patients with chronic kidney disease, as it is mainly excreted into bile, and reduced glomerular filtration does not affect its effectiveness [17]. Statins work by blocking HMG-CoA reductase, which lowers the production of mevalonatet and thus cholesterol. Statins reduce the mortality in patients with cardiovascular disease, as they lead to the decrease in serum cholesterol, but they have also shown pleiotropic effects [18]. Nevertheless, long-term statin therapy may contribute to the development of type 2 diabetes by reducing insulin secretion from the pancreatic beta cells. Therefore, it seems reasonable to closely monitor carbohydrate metabolism during the lipid-lowering therapy. However, the benefit of prolonging patients’ lives resulting from the use of statins significantly exceeds the risk associated with complications, so it is not surprising that they are widely used in cardiovascular diseases or that the new potential applications of statins are sought [19].

It has been shown that taking lipophilic statins such as simvastatin, atorvastatin, lovastatin, fluvastatin and pitavastatin, which can penetrate into cancer cells, is associated with a better prognosis in patients with breast cancer. On the other hand, improved survival in patients with hepatocellular carcinoma, colon cancer or prostate cancer is visible after the use of any statin [20]. The broad spectrum of the action of statins includes their effects on the proliferation, migration and survival of cancer cells by regulating the Rho factor, resistance to audiogenic seizures (Ras) and Rac-like guanosine triphosphate (GTP)-binding protein 1 (Rac) proteins, but also by inhibiting cancer cell growth modulated by specific pathways [21]. Co-administration of fluvastatin and vorinostat successfully induced apoptosis and reduced renal cancer growth in vitro and in vivo through the activation of 5’ adenosine monophosphate-activated protein kinase (AMPK), histone acetylation and cell stress induction [22]. Another study proved that simvastatin deregulated mutant p53 protein, activating the caspase-dependent apoptotic pathway and thus reducing the mobility of p53-mutated lung cancer cells [23]. In addition to their angiostatic effect, statins inhibit the synthesis of cytokines, including interleukin (IL-) IL-1β, IL-6, IL-8 and tumor necrosis factor alpha (TNF-α). The set of cytokines in the cluster of cancer cells stimulates them to proliferate and reorganize the spatial network of blood vessels, which promotes metastasis [24,25]. In studies conducted in mice transplanted with human breast cancer cells (MDA-MB-237), administration of cerivastatin to the tumor tissue significantly reduced the cross-linking of blood vessels of pathological tissue [26].

The integrity and permeability of blood vessels play an important role in metastasis. Statins have been shown to induce the expression of endothelial cadherins (including VE-cadherin), which are responsible for tight intercellular junctions. Increasing the number of cadherins is associated with strengthening vessels tightness, which limits the expansion of cancer cells in the body [27,28]. Moreover, statins inhibit the reprogramming of cancer cells into cancer stem cells responsible for metastasis and tumor immunity by reducing the transcriptional activity of the Yes1-associated transcriptional regulator (YAP1) and fafazzi (TAZ) and inhibiting geranylgeranyl pyrophosphate (GGPP) production [8].

Statins also regulate autophagy, which is triggered as a cellular defense mechanism when the elimination of toxic stress-inducing components or elements can ensure cell survival. It has been shown that autophagy can participate in both tumor suppression and its progression. On the one hand, it reduces the viability of cancer cells, is extremely important in the early stages of cancer promotion and represents a critical mechanism, and on the other hand, it stimulates aging processes caused by oncogenes. The tumor promoting effect of autophagy is mainly exerted in the later stages of cancer development. The dynamic proliferation of cancer cells requires large energy resources, which autophagy provides by recycling cell substrates [29]. Moreover, it promotes the survival of cancer cells under stressful conditions, such as tumor hypoxia and nutrient deficiency [30]. Simvastatin-induced autophagy has been reported in rhabdomyosarcoma cells [31]. It should be emphasized that autophagy also plays an important role in inducing resistance to chemotherapy [32].

**Figure 1:**
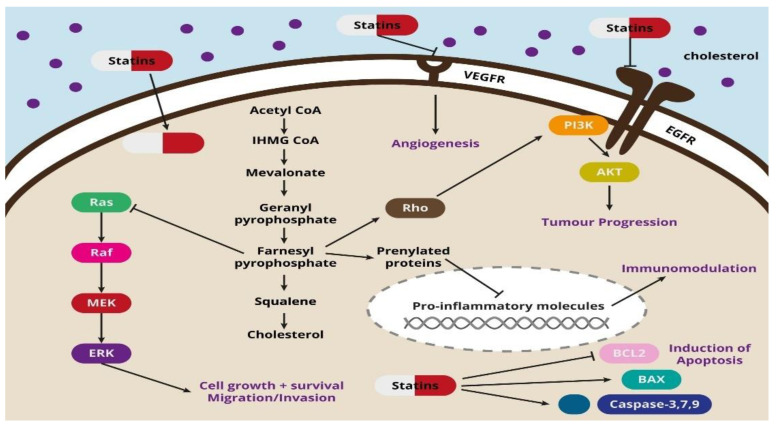
Figure representing the mevalonate pathway and the suggested molecular mechanisms of the action of statins. VEGFR, receptors for vascular endothelial growth factor; CoA, coenzyme A; Ras, resistance to audiogenic seizures; Raf, Raf oncogene, MEK, mitogen-activated protein kinase kinase; EGFR, epidermal growth factor receptor; ERK, extracellular-signal-regulated kinase; PI3K, phosphoinositide 3-kinase; AKT, protein kinase B; BCL2, B-cell lymphoma 2; BAX, BCL2 associated X, apoptosis Regulator.

An interesting phenomenon is that statins can also induce ferroptosis, which is another type of programmed cell death. This process is triggered by metabolic changes, and it is characterized by iron overload, accumulation of reactive oxygen species and lipid peroxidation, which can be used in statin therapy of cancer cells resistant to chemotherapy [32]. Another process induced by statins is pyroptosis, accompanied by cell swelling, rupture, lysis and, as a result, the release of pro-inflammatory cytokines. Studies have shown that pyroptosis is closely related to the development of various diseases, including cancer. Pyroptosis-inducing molecule gasdermin D (GSDMD) may be a promoter of gastric cancer cell proliferation, which has been proven both in in vitro and in vivo studies [33]. The potential use of statin-induced pyroptosis and its importance in the anticancer aspect requires further research and explanation. The potential mechanisms of the action of statins are shown in Figure 1.

Based on model breast cancer and malignant glioma cell lines, it has been proven that statins reducing the invasiveness of pathological cells act through changes in the level of protein phosphorylation and the activation of protein kinases in Rho/Rho kinase (ROCK) and Rho/Focal adhesion kinase (FAK)/Protein kinase B (Akt) pathways. As a consequence, signal flow is blocked at the FAK and Akt kinase level, possibly contributing to the activity changes of transcription factors Jun proto-oncogene, AP-1 transcription factor subunit (AP-1), β-catenin and nuclear factor kappa B (NF-kB), mediating the expression of genes involved in the multi-level cascade of metastatic events [8].

Epigenetics refers to all changes in the functioning of genes that are inherited without interfering with the DNA sequence. Interestingly, these changes are reversible during embryonic development as well as during cell proliferation [34]. Epigenetic modifications include DNA methylation, post-translational histone modifications, chromatin remodeling and nucleosome positioning. Epigenetic changes may also be associated with non-coding RNA molecules, which include microRNAs (miRNAs). Disturbances in the inheritance of expression patterns result in the abnormal activation or inhibition of signaling pathways, leading to the development of diseases, including cancer [35]. Histone deacetylases and their inhibitors (HDAC) are also important in the context of finding new cancer treatment strategies.

Xie et al. observed HDAC1 overexpression in human hepatocellular carcinoma and liver cancer cell lines. Its knockdown resulted in the induction of p21 and p27 expression and the suppression of cyclin D1 and CDK2 expression. As a consequence, the G1/S cell-cycle transition was impaired, leading to autophagic cell death [36]. Similarly, Fan et al. reported that HDAC5 knockdown in hepatocellular carcinoma promoted apoptosis and simultaneously inhibited tumor cell growth, due to the increase in caspase 3, p53 and Bax expression and the induction of the G1 phase cell-cycle arrest [37]. Statins are also involved in the regulation of the histone acetylation level. As previously mentioned, depending on the cancer type and experimental model, the actions of statins can have different effects.

Otahal and his team proved that fluvastatin or pitavastatin combined with erlotinib led to apoptosis in human lung adenocarcinoma cells with a mutated or overexpressed epidermal growth factor receptor (EGFR) [38,39,40]. In cancer studies, it has been shown that statin use can significantly reduce mortality in women [41]. In turn, Huang et al. reported that lovastatin inhibits cell proliferation and induces apoptosis through the regulation of p21 expression, leading to p53 overexpression and the suppression of survivin expression, eventually inducing cell death [42]. In this case, lovastatin exhibits antitumor activity through the survivin cascade in the LKB1-AMPK-p38MAPK-p53 pathway; however, describing the exact mechanism requires further research and is likely dependent on the type of tumor and patient’s genetic background. Transcription factor c-Myc, which mediates the regulation of key biological functions, is overexpressed in 31–64% of medulloblastomas [43]. Takwi et al. proved that lovostatin decreased the expression of mi-R-33b, a specific inhibitor of c-Myc, leading to reduction in the proliferation potential of cancer cells [44].

## 3. Cancer In Vitro Data and Statins

Experimental studies have shown that low doses (0.005–0.01 μmol/L) of cerivastatin and atorvastatin stimulate vascular endothelial cells to create new vascular structures [45]. On the other hand, higher doses (0.05–1 μmol/L) of statins inhibit the proliferation of endothelial cells, reducing the angiogenic capacity of factors that form new vascular networks and inducing apoptosis [45]. Huang et al. reported that statin can inhibit proliferation and induce apoptosis in breast cancer [42]. On the other hand, Bridgeman et al. noted that statins did not affect HDAC activity in the following cells: HepG2 human hepatocellular carcinoma cells, MDA-MB-231 human breast carcinoma cells, BRIN-BD11 rat insulinoma cells and THP-1 human leukemic monocytes [46]. Ishikawa et al. showed that simvastatin use, together with HDAC class II inhibitor (MC1568), causes a synergistic antiproliferative effect in colorectal cancer [31]. In addition, the antitumor effect of statins may be enhanced when HDAC1 is inhibited in cancer cell lines (CAL-27 and SACC-83) [47]. Iannelli et al. investigated the antitumor effect of valproic acid and simvastatin in prostate cancer cell lines PC3, 22Rv1, DU145, DU145R80 and LNCaP. They noticed that this combination sensitized metastatic castration-resistant prostate cancer cells to docetaxel [48]. Lin et al. confirmed the synergistic effect of mevastatin and HDAC inhibitor (LBH589) in triple-negative breast cancer. Their combination led to the activation of LKB1/AMPK signaling, cell cycle arrest in G2/M phase, increased apoptosis and lower tumor volume in xenografted mice [49]. These results are promising for further studies identifying new cancer therapies. The use of a combination of statins and HDAC inhibitors may therefore provide a much better result than using them alone [39].

The Increased proliferation of cancer cells is caused by elevated cholesterol levels in these cells [50]. In vitro studies on medulloblastoma lines showed that simvastatin inhibits HMC-CoA reductase, thereby affecting the inhibition of metalloproteinase secretion via GGPP [8,51]. It is worth adding that HMG-CoA reductase is extremely important in maintaining cell viability, and its inhibition induces apoptosis and negatively affects cell proliferation. GGPP can serve as a substrate for protein prenylation or as a precursor for the synthesis of other metabolites, such as coenzyme Q or dolichols. In recent years, it has become clear that different types of cancer cells are dependent on GGPP synthesis [52]. Significant changes in the MMP-9 levels were observed in in vitro cultured human breast cancer cells [20]. Fluvastatin and simvastatin also reduced the release of MMP-9 in human and murine macrophages [53,54]. In turn, in studies of human microvascular endothelial cells (HMEC-1), cerivastatin dose-dependently reduced or completely blocked the expression or synthesis of MMP-2 [55].

### Mechanism of Action

In vitro pro-apoptotic activity has been documented in studies on cancer cell lines, including cancer of the cervix, pancreas, thyroid gland, prostate, colon, breast and larynx, as well as multiple myeloma or leukemia [28,29,30,31,32,33,34,35,36]. Importantly, statins activated various molecular events that promote apoptosis, depending on the type of cancer cell. Statins also affects the expression and activity of cyclins and cyclin-dependent kinases (CDKs) and their inhibitors. This leads to a blockage of the cell cycle at two of its critical points: G1/S and G2/M, thus inhibiting cell division. Under conditions where the statin dose is adequate and there is an imbalance between pro- and anti-apoptotic proteins, statins induce apoptosis [56,57,58,59,60,61,62,63].

One of the key mechanisms of the antitumor activity of statins is also the angiostatin effect in neoplastic lesions [64,65]. It is based on the expression modulation of anti- and pro-angiogenic factors, including VEGF, FGF and IGF-1, leading to the inhibition of angiogenesis and, as a result, reduction in metastatic capacity of cancer cells [64,66,67]. The dualistic nature of statins in terms of their role in the formation of blood vessels is observed in scientific literature, where the vast majority of studies report the anti-angiogenic effects of statins on cancer cells; however, a few demonstrate their ability to stimulate endothelial cell division and angiogenesis [27,65,68].

The clear presence of metalloproteinases in tumor masses, through the remodeling of extracellular matrix and basement membrane, has an unfavorable prognosis [69]. Organizational changes in the cell cytoskeleton, destabilization of actin filaments or changes in morphology favoring apoptosis are also a consequence of statins’ influence on cancer cells. The great potential of statins in terms of their use as drugs delaying progression and expansion of cancer has been proven in the course of experimental work [20,51,70].

## 4. Animal Cancer In Vivo Data and Statins

Studies have shown that statins, in this case simvastatin, induce the phosphorylation of endothelial nitric oxide synthase, inhibiting apoptosis and promoting Akt-dependent angiogenesis in the ischemic limbs of normocholesterolemic rabbits [71]. Lipophilic statins have higher cytotoxic potential and are more pro-apoptotic than hydrophilic statins, and therefore may be more beneficial in cancer treatment [67].

In in vivo studies in mice, pitavastatin was injected intraperitoneally, which inhibited the growth of subcutaneous glioma cells [72]. Simvastatin tested in a mouse model in which a human lung cancer xenograft was introduced inhibited tumor growth and bone metastasis, which occurred with a simultaneous reduction in MAPK/ERK activity [57]. The inhibition and delay of tumor growth was also observed in a pancreatic cancer xenograft in mice treated with a combination of gemcitabine and fluvastatin [73]. Advanced clinical trials are ongoing, with a large amount of promising in vitro and in vivo data on the efficacy of statin therapy in several different cancer models [8,74].

## 5. Human Cancer In Vivo Data and Statins

### 5.1. Epidemiology

The importance of statins in the context of cancer has been discussed in many studies, the results of which can be contradictory [75,76,77,78,79,80,81,82,83,84,85,86,87]. Since the first reports in the late 1990s on the effect of statins on cancer progression, numerous in vitro and in vivo studies have been carried out to determine their antitumor properties against solid tumors of liver, brain and lungs, as well as in diffuse cancers such as multiple myeloma or leukemia [75]. Due to the well-established safety profile of statins, conducting clinical trials on their use in oncology is much cheaper than the cost of the development of new drugs [8].

### 5.2. Meta-Analysis

One of the larger meta-analyzes carried out recently included a study on the use of statins in oncology in the Danish population [8,88]. Meta-analysis showed that statin use can reduce cancer mortality by about 40% [89,90]. In turn, another meta-analysis showed that concomitant treatment with statins and standard anticancer drugs did not significantly improve the survival rate or progression-free survival of patients with advanced cancer and prognosis of less than 2 years [91]. In a retrospective cohort study (2006–2014), researchers found that use of any type of statin was associated with a reduction in the mortality of pancreatic cancer patients [8]. There is even evidence to suggest that statins may potentially increase the risk of certain types of cancer, such as prostate cancer and melanoma [92]. The ambiguity of the results makes further research on the effects of statins on cancer treatment necessary.

The antitumor effect of statins is largely related to their ability to induce apoptosis, targeting cancer cells with high selectivity [50]. Mechanisms underlying their pro-apoptotic action are related to inhibition of the cholesterol biosynthetic pathway, with the mevalone pathway (MVA) appearing to be of major importance. Secretion reduction in the intermediate metabolites of the MVA pathway (via statins) contributes to the appearance of morphological changes, which are characteristic of programmed cell death [72,93].

### 5.3. Observational Studies

One of the largest observational studies on statins use in cancer prevention is an analysis based on the Danish Cancer Registry. In a group of 300,000 patients, it was reported that statins significantly reduced cancer incidence (OR 0.86; 95% CI 0.78–0.95) [76]. Kuoppala et al., based on their meta-analysis, which included 25 observational studies and 17 randomized trials, found that the risk of developing cancer did not differ significantly in the group taking statins from the control group (OR 0.96; 9% CI 0.72–1.20) [77]. Interestingly, another meta-analysis based on 20 case–control studies showed a beneficial effect of statins in the prevention of hyperproliferative diseases [78]. However, the results of the 2019 meta-analysis, which examined data collected from meta-analyzes of randomized controlled trials (RCTs) and observational studies assessing the effect of statins on the risk of cancer, should be taken into account. These authors also analyzed the evidence strength from which the conclusions of 43 studies were drawn. Based on the conventional method of assessing the significance of meta-analysis (*p* < 0.05), the beneficial effect of statins was noted in 10 out of 18 examined tumors. However, when the evidence strength was taken into account, there was no significant effect of statin use on cancer risk. The authors of this study emphasized that the benefits of pharmacotherapy with statins should be evaluated individually in each case [89].

### 5.4. Randomized Controlled Trials (RCTs)

Long-term RCTs play an important role in research [94]. In one, 20,000 patients diagnosed with cancer were randomized into two groups, one of which received 40 mg of simvastatin and one which received a placebo. A decrease in total cholesterol and number of cardiovascular events was observed in the group receiving the drug compared to placebo group [95]. Interestingly, an 11-year follow-up, which included 17,519 patients out of the previously mentioned 20,000 [14] revealed no significant relationship between statin use and cancer risk and cardiovascular events. However, it was found that low-density lipoprotein (LDL) concentration was reduced by 1 mmol/L during the observation period [96]. On the other hand, a meta-analysis of 26 randomized trials involving 86,936 participants, including 6662 with diagnosed cancer and 2407 deaths due to cancer, did not show that statin use had any effect on morbidity and survival, regardless of the cancer type [97,98]. The observations from the Cholesterol Treatment Trialists’ (CTT) study are interesting. A total of 27 RCTs with 174,149 participants were analyzed. Again, statin use has not been shown to significantly affect cancer risk or survival (OR 1.00, 95% CI 0.96–1.04, OR 0.99, 95% CI 0.93–1.06, respectively) [98,99,100,101]. Randomized studies demonstrated the efficacy of statin use in the adjuvant treatment of hormone-dependent breast cancer with improved disease-free survival (HR 0.79; 95% CI 0.66–0.95). Although the conducted meta-analyzes do not show a clear efficacy of statins in short- and medium-term follow-ups in the context of improved prognosis in cancer patients, the available data may suggest the potential benefits of long-term care. It seems that substances that have been known for years, such as statins or metformin, may turn out to be interesting in terms of their use as adjuvants for standard oncological treatment or in preventive medicine, and their great advantage is their low price and well-known and predictable side effects. However, confirming their effectiveness requires well-designed studies [102]. There are also ongoing clinical trials evaluating the efficacy and safety of beta-ketoacyl reductase inhibitor (TVB-2640) in non-small cell lung cancer, respectable colon cancer and HER2-positive breast cancer. In obese patients, this compound inhibited hepatic de novo lipogenesis, and in glioblastoma patients, when combined with bevacizumab, it prolonged life expectancy compared to historical controls. Prostate cancer is also characterized by a high rate of de novo fatty acid synthesis, but it is not currently tested for the use of TVB-2640 [103]. In turn, in patients with lung cancer, they can inhibit epithelial–mesenchymal transition (EMT) in a p53 mutation-dependent manner [104]. Studies indicate that statins increase histone H3 and H4 acetylation as well as inhibit class I and II HDACs [105].

### 5.5. Pharmacoepidemiological Studies

In recent years, due to the increasing access to population data sets deposited in repositories, the number of pharmacoepidemiological studies is growing [106,107,108]. Such analyzes allow us to assess the real effect of drugs, including statins, both in the prevention and treatment of cancer by simultaneously considering and comparing many variables [106]. An example of such an analysis is the AspECT study conducted at 84 centers in the UK and 1 in Canada, which aimed to assess the effectiveness of esomeprazole and aspirin in preventing esophageal adenocarcinoma development in patients with Barrett’s esophagus. This study was justified due to the available pharmacoeconomic evidence, and the chemopreventive effect of treatment with these two drugs in the context of esophageal cancer in patients diagnosed with Barrett’s esophagus was confirmed [109]. It seems that this type of analysis should be approached with caution, as drug efficacy studies based on population registries may be burdened with errors, e.g., methodological, lack of randomization and bias [110,111]. Soni et al., in a meta-analysis, compared the results obtained from population studies with RCTs focused on the assessment of cancer treatment effectiveness but did not find any agreement between them [112]. Dickerman et al. revealed that in many observational studies, people using statins have a significantly lower risk of developing cancer than in the meta-analyzes of randomized trials. They reported no association between statin use and cancer risk. It is therefore important in epidemiological studies based on the analysis of large data set to strive for such analyzes to find the relationship between a specific treatment and its effects [113,114,115,116].

### 5.6. Mendelian Randomization

Analyzes taking into account Mendel’s randomization methods, examining the influence of genetic variants found in populations on the occurrence of a specific effect, are an important type of research. It is based on the well-established knowledge that the polymorphism of the relevant genes is related to specific behaviors. The study of genetic variants avoids confounding factors and errors associated with conducting observational studies. The inclusion of this randomization has opened an era of genomic research. It is used not only to identify links between gene groups and disease occurrence but can also be an alternative or complement to RCTs aimed at establishing a cause-and-effect relationship between exposure to a given factor and disease occurrence. According to Mendel’s second law, gene variants (polymorphisms) are randomly distributed among reproductive cells (sperm and ova), and therefore natural randomization occurs. These phenomena underlie the term “Mendelian randomization” [104,105,106]. For this reason, studies on the effect of statins on carcinogenesis focus on the polymorphisms of genes involved in cholesterol biosynthesis, which are therapeutic targets of statin therapy. Orho-Melander et al. assessed 26–41 single nucleotide polymorphisms (SNPs) of genes encoding proteins involved in the synthesis of cholesterol and its fractions, as well as the determined impact of rs12916 HMGCR polymorphism, encoding protein targeted by statins, on the risk of cancer development. They confirmed the relationship between serum triglyceride concentration and cancer risk and that HMGCR polymorphism may be associated with the risk of prostate and breast cancer [104]. In turn, when the significance of s12916 HMGCR was assessed in men with prostate cancer (n = 22,773) compared to the control (n = 23,050), the studied relationship was relatively small (OR 0.97; 95% CI 0.94–1.00; *p* = 0003) and did not correlate with the advancement of proliferative changes [105]. Additionally, another study analyzed three HMGCR polymorphisms in terms of breast cancer risk (n = 122.977) compared to control (n = 105.974). HMGCR gene inhibition has not been shown to significantly reduce the risk of developing breast cancer (OR 0.86; 95% CI 0.73–1.02; *p* = 0.09) [106]. On the other hand, genomic studies revealed a significantly lower risk of ovarian cancer, both with and without BRCA1/2 mutation carriers, in the statin group (OR 0.60; 95% CI 0.43–0.83; *p* = 0.002 vs. OR 0.69; 95% CI 0.51–0.93; *p* = 0.01) [107]. Therefore, the inclusion of functional genomics studies is important, due to the fact that they indicate new, potential therapeutic targets, to help us understand different clinical responses in patients using the same drug, contributing to the personalization of treatment [108,109]. Table 1 summarizes observations indicating the usefulness of statins in cancer prevention.

### 5.7. Mechanism of Action

The antitumor effect of statins is largely related to their ability to induce apoptosis, targeting cancer cells with high selectivity [50]. Mechanisms underlying their pro-apoptotic action are related to the inhibition of the cholesterol biosynthetic pathway, with the mevalone pathway (MVA) appearing to be of major importance. The secretion reduction of the intermediate metabolites of the MVA pathway (via statins) contributes to the appearance of morphological changes, which are characteristic of programmed cell death [72,93]. 

Orho-Melander et al. assessed 26–41 single nucleotide polymorphisms (SNPs) of genes encoding proteins involved in the synthesis of cholesterol and its fractions, as well as the determined impact of the rs12916 HMGCR polymorphism, encoding protein targeted by statins, on the risk of cancer development. They confirmed the relationship between serum triglyceride concentration and cancer risk, and that HMGCR polymorphism may be associated with the risk of prostate and breast cancer [117]. In turn, when the significance of s12916 HMGCR was assessed in men with prostate cancer (n = 22,773), compared to control (n = 23,050), the studied relationship was relatively small (OR 0.97; 95% CI 0.94–1.00; *p* = 0.003) and did not correlate with the advancement of proliferative changes [118]. Additionally, another study analyzed three HMGCR polymorphisms in terms of breast cancer risk (n = 122.977) compared to the control (n = 105.974). HMGCR gene inhibition has not been shown to significantly reduce the risk of developing breast cancer (OR 0.86; 95% CI 0.73–1.02; *p* = 0.09) [119]. On the other hand, genomic studies revealed a significantly lower risk of ovarian cancer, both with and without BRCA1/2 mutation carriers, in the statin group (OR 0.60; 95% CI 0.43–0.83; *p* = 0.002 vs. OR 0.69; 95% CI 0.51–0.93; *p* = 0.01) [120]. Therefore, the inclusion of functional genomics studies is important due to the fact that they indicate new, potential therapeutic targets, which help us to understand different clinical responses in patients using the same drug, contributing to the personalization of treatment [123,124]. Table 2 summarizes observations indicating usefulness of statins in cancer prevention.

## 6. Conclusions

Despite the recent development of diagnostics and therapy, cancer remains an ongoing problem. Studies are carried out to identify novel molecular markers as well as therapeutic strategies to diagnose cancer as early as possible and implement appropriate treatment. Attention is also paid to factors lowering the risk of cancer as well as preventing it. One of them may be the use of statins. 

Statins exhibit pleiotropic activity, which in the case of cancer, may be manifested by the induction of cancer cell apoptosis with high selectivity. Research shows that statins can also induce other types of programmed cell death, including ferroptosis. In addition, their participation in the regulation of autophagy is indicated. Therefore, the use of statins in the treatment of cancer is promising. Interestingly, the combination of statins together with different drugs, including histone deacetylase inhibitors, seems to give better results than using them alone. This review summarizes the in vitro and in vivo studies, meta-analyzes, observational studies and randomized controlled trials which help us to understand the crosstalk between statin use and cancer.

## Figures and Tables

**Table 1:** Usefulness of statins in cancer prevention.

Type of Study	Authors	Number of Cases (n)	Results (95% Confidence Interval)
Pharmaco-epidemiology using a target trial design	Dickerman, Nature Medicine [113] (2019)	n = 28,408 cases	Cancer-free survival difference−0.5% (−1.0–0.0%)
Mendelian randomization	Orho-Melander, Int J Epidemiol [117] (2018)	n = 6528	HR equivalent to 0.07 mmol/L LDL lowering 0.99 (0.95–1.02)
	Bull, Cancer Medicine [118] (2016)	n = 22,773 prostate	OR 0.97 (0.94–1.00)
	Nowak, Nature communications [119] (2018)	n = 122,977 breast	OR equivalent to 1 mmol/L LDL lowering 0.86 (0.73–1.02)
	Yarmolinsky, JAMA [120] (2020)	n = 25,509 ovarian n = 3887 BRCA 1/2	OR 0.6 (0.43–0.83) OR 0.69 (0.51–0.93)
In vivo evidence at biologically relevant doses	Newman, JAMA [121] (1996)	Review of rodent carcinogenicity studies	Statins increase incidence of cancer
Meta-analyses of epidemiological data	Kuoppala, Eur J Cancer [77] (2008),	42 differing study designs n = 67,432 cases	RR 0.96 (0.72–1.12)
	Taylor, Eur J Cancer Prevention [65] (2008)	20 case–control n = 100,129	OR 0.71 (0.56–0.89)
Meta-analyses of RCTs	Dale, JAMA [97] (2006)	26 RCTs n = 6662 cases	OR 1.02 (0.97–1.07)
	Kim, Indian J Cancer [98] (2017)	21 RCTs n = 32,615 on statin	RR 0.97 (0.92–1.02)
Individual patient data from RCTs	CTT, Lancet [99] (2012), PLOS [100] (2012), Lancet [101] (2010), Lancet [122] (2019)	27 RCTs n = 175,000	Incidence RR 1.00 (0.96–1.05) Mortality RR 1.00 (0.93–1.08)

OR, odds ratio.

**Table 2:** Molecular medicine aspect of the statin–cancer association.

Combination	Pathology Association	Pathways	References
**In Vitro**			
mevastatin and HDAC inhibitor (LBH589)	triple-negative breast cancer	LKB1/AMPK signaling, cell cycle arrest in G2/M phase, increased apoptosis, lower tumor volume	[49]
simvastatin	medulloblastoma lines	inhibits HMC-CoA reductase, thereby affecting inhibition of metalloproteinase secretion via GGPP	[8,51]
**In Vivo (Animals)**			
simvastatin	limb ischemia	induce phosphorylation of endothelial nitric oxide synthase, inhibiting apoptosis and promoting Akt-dependent angiogenesis	[71]
	human lung cancer xenograft	inhibited tumor growth and bone metastasis- reduction in MAPK/ERK activity	[73]
pitavastatin	subcutaneous glioma cells	inhibited growth	[72]
gemcitabine and fluvastatin	pancreatic cancer xenograft	inhibition and delay tumor growth	[74]
**In Vivo (Humans)**			
statins	cancer cells	pro-apoptotic action are related to inhibition of cholesterol biosynthetic pathway, with mevalone pathway (MVA)	[72,93]

## Data Availability

All data generated or analyzed during this study are included in this published article.
